# Draft genome sequence of benzo[a]pyrene degrading *Bacillus altitudinis* strain AR19 isolated from Digboi oil refinery (India)

**DOI:** 10.1128/mra.00957-24

**Published:** 2025-03-13

**Authors:** Abhisek Dasgupta, Ratul Saikia, Bibhuti B. Kakoti, Pratap Jyoti Handique

**Affiliations:** 1 Centre for Biotechnology and Bioinformatics, Dibrugarh University, Dibrugarh, India; 2 Biological Sciences and Technology Division, CSIR-North East Institute of Science and Technology, Jorhat, India; 3 Department of Biotechnology, Gauhati University, Guwahati, India; Department of Biological Sciences, Wellesley College, Wellesley, Massachusetts, USA

**Keywords:** whole-genome sequencing

## Abstract

*Bacillus altitudinis* AR19, isolated from the Digboi oil refinery (India), has a genome size of 3,630,000 bp with a G+C content of 42.45%. The genome encodes 3,755 protein-coding genes, including those for ring-cleaving dioxygenases and biofilm formation. These genes likely play crucial roles in the bacterium’s survival in hydrocarbon-enriched environments.

## ANNOUNCEMENT

Benzo[a]pyrene degrading *Bacillus altitudinis* strain AR19 was isolated from soil sample collected from Digboi oil refinery (27°23′27″N 95°37′07″E) situated in India. Sixteen polycyclic aromatic hydrocarbons (PAHs) were recognized by the US Environmental Protection Agency as significant environmental pollutants (1), out of which benzo[a]pyrene (B[a]P) is one of them. B[a]P, which belongs to high molecular weight PAHs, is also considered to be a potent carcinogen to humans, as recognized by the International Agency for Research on Cancer (2). *B. altitudinis* has been found to degrade aromatic compounds found in hydrocarbon pollutants (3). *B. altitudinis* species belongs to the rod shape, gram-positive bacteria classified under *Firmicutes* (4). It was first reported to be isolated from air samples collected from the stratosphere. It has diverse habitat (4). *B. altitudinis* strain AR19 was isolated using the serial dilution method (at 37°C) and screened against benzo[a]pyrene (Sigma-Aldrich) using spray plate technique in Bushnell Haas Agar (HIMEDIA) (5). AR19 was found to be positive against benzo[a]pyrene. Overnight culture of 48 hours of *B. altitudinis* strain AR19 was used to extract the genomic DNA. GeNei Bacterial DNA purification kit (Genei Laboratories Private Limited, Bengaluru, India) was used for DNA extraction, and isolation of the genomic DNA of *B. altitudinis* strain AR19 was done using the manufacturer’s protocol. The genomic DNA was quantified by BioSpectrometer (Eppendorf, Germany).

The genomic DNA of *B. altitudinis* strain AR19 was outsourced to AgriGenome Labs Pvt Ltd (India) for sequencing. The library was prepared using the Illumina DNA Prep kit (Illumina, California, USA) following the manufacturer’s protocol. Sequencing was done on the Illumina HiSeq 2500 platform. Paired-end reads were generated with read length of 250 bp. The total reads generated were 4,199,382. Quality check of the raw reads was done by the FastQC tool (6). The adapter sequence was removed by Cutadapt v1.8 (7). Fastp was used to join the trimmed reads (8). Kmergenie was used to check the kmers for *de novo* assembly, which was done in SPAdes v. 3.13.1 (9, 10). The genome assembly was evaluated using Quast (11). The genome assembly was done using k-mer size of 67, 69, 71, 73, 75, and 77. QUAST reported predicted that k-mer of size 73 was the best assembly with the largest contig size of 299,197 bp and N50 equal to 94,649 bp and the total number of contigs equal to 85. The assembly size was 3,630,000 bp. GTDB-Tk (Genome Taxonomy Database) tool was used for phylogenetic analysis (12). The AR19 genome also showed closest reference with *B. altitudinis*. The genome was annotated using the NCBI Prokaryotic Genome Annotation Pipeline (13). For all the software used in this study, default parameters were used except where otherwise noted.

The genome of *B. altitudinis* strain AR19 encodes 3,849 total number of genes, out of which 3,755 were protein-coding genes and 72 RNA genes. Notable proteins include catechol 2,3-dioxygenase, mechanosensitive channel and putative ring-cleaving dioxygenase (MhqO). These proteins may contribute to the strain’s resistance to benzo[a]pyrene (14–16).

## Data Availability

The sequencing data have been deposited in the NCBI database The raw reads and assembly can be accessed using the accession numbers SRP540094 and QAWE00000000, respectively.
